# Alternative Lengthening of Telomeres (ALT) in Pancreatic Neuroendocrine Tumors: Ready for Prime-Time in Clinical Practice?

**DOI:** 10.1007/s11912-021-01096-w

**Published:** 2021-07-16

**Authors:** Claudio Luchini, Rita T. Lawlor, Samantha Bersani, Caterina Vicentini, Gaetano Paolino, Paola Mattiolo, Antonio Pea, Sara Cingarlini, Michele Milella, Aldo Scarpa

**Affiliations:** 1 Department of Diagnostics and Public Health, Section of Pathology, University and Hospital Trust of Verona, 37134 Verona, Italy; 2 ARC-Net Research Centre, University and Hospital Trust of Verona, 37134 Verona, Italy; 3 Department of Surgery, University and Hospital Trust of Verona, Verona, Italy; 4 Department of Medicine, Section of Oncology, University and Hospital Trust of Verona, Verona, Italy

**Keywords:** Pancreatic neuroendocrine tumors, PanNET, ALT, DAXX, ATRX

## Abstract

**Purpose of Review:**

Alternative lengthening of telomeres (ALT) is a telomerase-independent mechanism used by some types of malignancies, including pancreatic neuroendocrine tumors, to overcome the issue of telomere shortening, thus supporting tumor growth and cell proliferation. This review is focused on the most important achievements and opportunities deriving from ALT assessment in PanNET onco-pathology, highlighting the most promising fields in which such biomarker could be implemented in clinical practice.

**Recent Findings:**

In pancreatic neuroendocrine tumors (PanNET), ALT is strongly correlated with the mutational status of two chromatin remodeling genes, *DAXX* and *ATRX*. Recent advances in tumor biology permitted to uncover important roles of ALT in the landscape of PanNET, potentially relevant for introducing this biomarker into clinical practice. Indeed, ALT emerged as a reliable indicator of worse prognosis for PanNET, helping in clinical stratification and identification of “high-risk” patients. Furthermore, it is a very specific marker supporting the pancreatic origin of neuroendocrine neoplasms and can be used for improving the diagnostic workflow of patients presenting with neuroendocrine metastasis from unknown primary. The activation of this process can be determined by specific FISH analysis.

**Summary:**

ALT should be introduced in clinical practice for identifying “high-risk” PanNET patients and improving their clinical management, and as a marker of pancreatic origin among neuroendocrine tumors.

## Introduction

Since somatic cells are unable to polymerize all DNA during physiological replication, each round of cell division determines telomere shortening [1,2]. This mechanism is the same in cancer cells, where it is used to support cell proliferation and the consequent tumor growth [3–5]. Thus, a tumor can achieve a remarkable replicative capacity also thanks to the activation of this telomere maintenance mechanism [3–5].

**Fig. 1:**
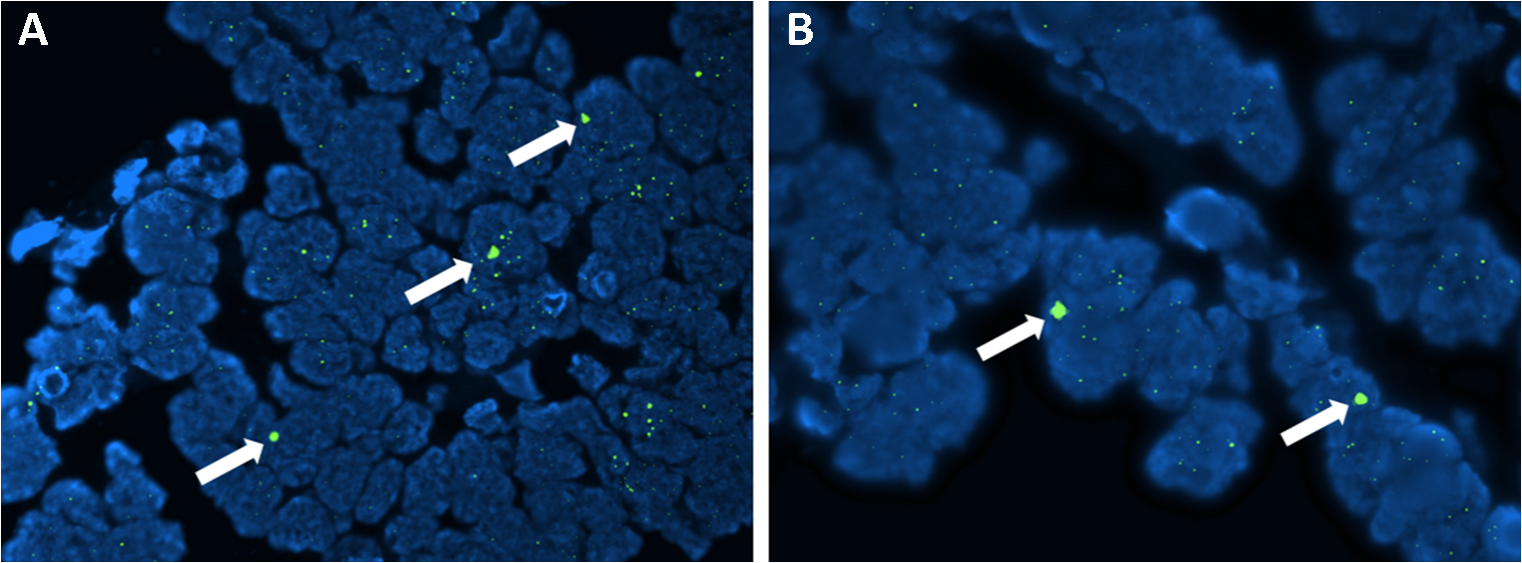
A classic example of a pancreatic neuroendocrine tumor with the activation of the ALT mechanism analyzed with telomere-specific FISH. The presence of large ultrabright telomere FISH signals (arrows) is the pictorial marker of the activated alternative lengthening of telomere mechanism (**A** ×4 original magnification; **B** ×10)

Physiologically, telomere shortening is counterbalanced by the specific activity of the enzyme telomerase, which is a DNA polymerase that uses reverse transcription of a RNA template to produce de novo synthesis of telomeric DNA [5]. Interestingly, some neoplasms do not overcome the issue of telomere shortening by using telomerase, but maintain telomere length with a different process, which is totally telomerase-independent and called alternative lengthening of telomeres (ALT) [6,7]. Differently from the telomerase-dependent mechanism, in ALT, the telomeric DNA is produced on the basis of a DNA template [6]. Indeed, the copy template can either be represented by the telomere itself, by a telomere on a sister chromatid, or even by another chromosome [8]. Due to these different mechanisms, ALT cells usually show long and heterogeneous telomeres with sub-nuclear formations, the ALT-associated promyelocytic leukemia bodies (APB), which comprise telomeric DNA and the telomere-specific binding proteins, also known as terminal restriction fragments (TRF) [8]. Thanks to these features, ALT status can be easily determined by specific FISH analysis (Fig. 1).

ALT has been detected in different tumor types, including soft-tissue sarcomas and brain tumors [9,10]. In soft-tissue sarcomas, a recent meta-analysis analyzing 551 patients with different types of sarcomas demonstrated that activation of the ALT mechanism was strongly associated with an increased risk of mortality (doubled risk of mortality, HR = 2.02) [11•]. The only exception was represented by osteosarcoma, in which ALT did not show a clear and reliable biologic significance. Notably, although ALT was associated with a higher mitotic count and tumor grade in this type of malignancies, it maintained its prognostic significance also using data from multivariable analysis, highlighting its prognostic relevance [11•].

In brain tumors, ALT appears as the major telomere maintenance mechanism in *IDH1*-mutated astrocytomas [12]. Furthermore, the activation of the ALT mechanism emerged as a common process associated to the metastatic behavior of primary medulloblastomas [13]. These findings highlight both the biological importance of ALT in tumor progression and its broad prognostic significance.

Interestingly, ALT has also been detected in pancreatic neuroendocrine tumors (PanNET), with important diagnostic and prognostic implications. Here we will analyze and discuss more in depth the most important findings related to ALT in PanNET, shedding light on its potential applications in clinical practice.

## ALT in Pancreatic Neuroendocrine Tumors

### ALT Is Strictly Associated with *DAXX*/*ATRX* Mutations

The first description of the presence of the ALT process in a significant proportion of PanNET was provided by Marinoni et al. in 2014 [14••]. The authors demonstrated that the activation of such process was strictly correlated with a malignant behavior, including a higher rate of distant metastasization. Notably, the authors also described a strong association of ALT with the mutational status of two chromatin remodeling genes, whose mutations are usually mutually exclusive: death domain-associated protein (*DAXX*) and α-thalassemia/mental retardation X-linked (*ATRX*) genes [14••]. These genes encode homonymous proteins that exert their function in the nucleus, by regulating the deposition of histone variant H3.3 during the assembly of peri-centromeric and telomeric chromatin [15]. Subsequent studies on the molecular landscape of PanNET confirmed the presence of ALT mechanism as a frequently biological process activated in this tumor type, and also its association with *DAXX*/*ATRX* mutations [16•, 17, 18••, 19]. In general, *DAXX* mutations were more commonly reported than *ATRX* mutations in PanNET [16•, 17, 18••, 19], whereas those affecting *ATRX* were markedly prominent in sarcomas [11•].

**Fig. 2:**
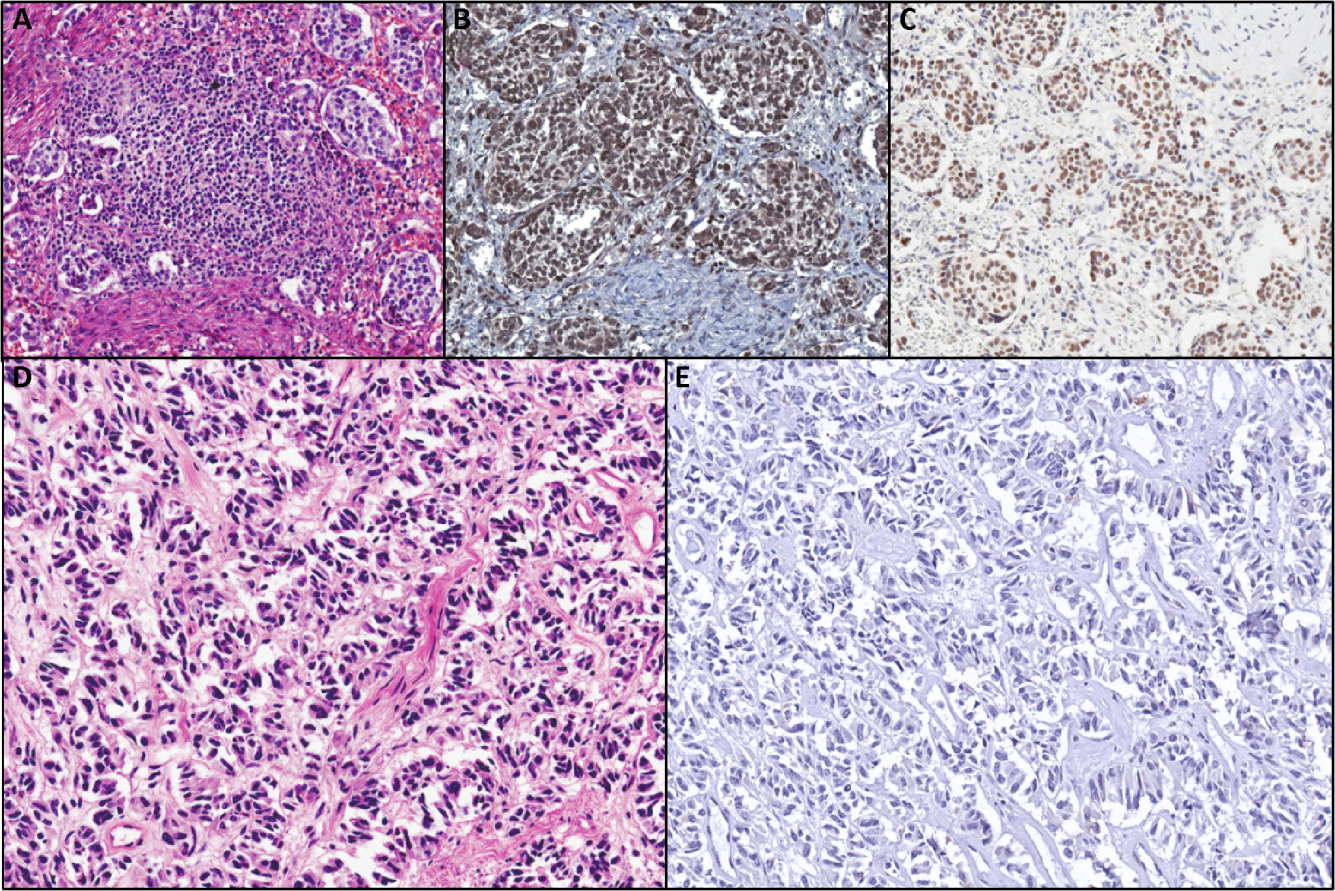
Representation of two paradigmatic cases of pancreatic neuroendocrine tumors, one with retained expression of DAXX/ATRX at immunohistochemistry (**A** hematoxylin-eosin, **B** DAXX immunostaining, **C** ATRX immunostaining; original magnification ×10) and one with DAXX loss (**D** hematoxylin-eosin, **E** DAXX immunostaining, highlighting loss of nuclear expression; original magnification ×20)

Of importance, the presence of *DAXX*/*ATRX* mutations can be studied not only with a direct molecular analysis but also with immunohistochemistry, which represents a reliable surrogate of their mutational status. Indeed, in the case of mutation, there is a total loss of expression of the corresponding protein at the immunohistochemical level (Fig. 2) [16•].

### ALT Is a Strong Prognostic Indicator in PanNET

In PanNET, the first description of the poor prognostic role of the activated ALT mechanism has been provided by Marinoni and colleagues that analyzed both cancer-specific and disease-free survival [14••]. These observations were further confirmed by different studies analyzing a broad spectrum of prognostic indexes [16•, 17, 18••, 20, 21••, 22•], highlighting the clinical value of this biomarker. In particular, the studies of Scarpa et al. that analyzed 102 PanNET and of Singhi et al. that analyzed 321 PanNET were the first reports that extensively analyzed and described the poor prognostic value of ALT also in G2 PanNET [18••]. Furthermore, Scarpa et al. showed that, in both PanNET G1 and G2, the activated ALT mechanism was also associated with a recurrent pattern of whole chromosomal loss, which typically involved specific chromosomes, comprising chromosomes 1, 2, 3, 6, 8, 10, 11, 15, 16, and 22 [18••]. All considered, ALT mechanism, *DAXX/ATRX* mutations, and specific chromosome loss compose a so-called ALT phenotype that demonstrated a poor prognostic impact in PanNET patients.

Kim et al. also correlated the ALT phenotype with a worse prognosis, and in particular with a reduced recurrence-free survival; furthermore, they highlighted that such feature was specifically correlated with specific histological parameters [21••]. Indeed, studying a cohort of 269 patients, they reported a strong association of ALT with tumor size, nodal metastasis, and vascular and perineural invasion [21••]. Notably, in their report, the authors analyzed ALT status also in G3 tumors, reporting the ALT activation in 2 of 8 high-grade pancreatic neoplasms. Interestingly, when limiting to patients with distant metastases, those with ALT-positive primary tumors had significantly better overall survival, further refining the knowledge on the prognostic role of ALT in PanNET.

Of interest is also a recent study of Pea and colleagues, which tested the prognostic power of ALT in a series of small PanNET, using 3 cm as an arbitrary threshold for considering a PanNET as a small lesion [16•]. As prognostic index, the authors analyzed the risk of liver metastasization. The cohort was composed of 87 small PanNET, and the presence of the ALT phenotype was confirmed as a poor prognostic indicator also in small lesions.

A last insight regarding the potential clinical value of ALT in PanNET has been provided by Hackeng et al. that focused their analysis on a cohort of 31 patients with insulinomas and found that ALT phenotype was strictly associated with the risk of developing liver metastasis [22•].

The fact that all findings on the poor prognostic impact of ALT are overlapping in different PanNET cohorts, including large and small lesions, G1 and G2 tumors, and non-functioning neoplasms and insulinomas, highlights that this biomarker can be used as a reliable prognostic indicator in PanNET patients. Its determination during the routine diagnostic workflow, above all for patients with small and non-functioning lesions, can help in selected patients for upfront surgery (ALT positive) vs. active surveillance (ALT negative).

### Other Insights on ALT in PanNET

A recent study assessed the feasibility of ALT determination on materials from fine needle aspiration (FNA), as well as of immunohistochemistry in determining DAXX and ATRX expression [23]. Investigating 65 PanNET, VandenBussche et al. established that both ALT status and DAXX/ATRX expression can be accurately performed on FNA specimens. The opportunity to determine ALT in small biospecimens has important implications for the introduction of these biomarkers in clinical practice. Thank to this possibility, indeed, ALT status and DAXX/ATRX expression may represent a decisive tool in the pre-surgical setting, helping in the identification of patients with “high-risk” lesions, thus improving PanNET clinical management.

In the case of neuroendocrine metastasis from unknown primary, which represents a non-negligible proportion of cases, Dogeas et al. indicated that the presence of the activated ALT mechanism could represent an important tool in supporting the pancreatic origin of a neuroendocrine tumor [24]. Indeed, analyzing a cohort of 90 gastro-enteropancreatic NET, they found that the specificity of ALT for detecting pancreatic origin was very high (96%). Also, this feature should be taken into account for implementing the possibility of ALT determination in clinical practice.

A last insight regards the possibility to predict the ALT status on the basis of radiological findings. McGovern and colleagues showed that some features identified at computed tomography were significantly associated with the ALT phenotype, such as tumor necrosis, vascular invasion, pancreatic duct dilation, and liver metastasization. These findings should be read in the context of multidisciplinary and precision medicine, where both pathology and radiology can integrate their information guiding the best clinical management of PanNET patients.

## Conclusions

The assessment of the ALT status can be very useful to improve the clinical practice for PanNET patients: (i) to stratify prognosis of resected patients, with immediate implications for patients management; (ii) in the pre-surgical setting, serving as an additional tool to select patients for surgical resection; and (iii) as a marker of pancreatic origin, improving the diagnostic workflow of patients presenting with neuroendocrine metastasis from unknown primary. Globally considered, these represent three major reasons that call for launching ALT determination in the PanNET clinical practice.

## Data Availability

All data are available in the manuscript.
